# Dexamethasone promotes mesenchymal stem cell apoptosis and inhibits osteogenesis by disrupting mitochondrial dynamics

**DOI:** 10.1002/2211-5463.12771

**Published:** 2019-12-30

**Authors:** Liang Ma, Xiaobo Feng, Kun Wang, Yu Song, Rongjin Luo, Cao Yang

**Affiliations:** ^1^ Department of Orthopedics Union Hospital Tongji Medical College Huazhong University of Science and Technology Wuhan China

**Keywords:** apoptosis, dexamethasone, mesenchymal stem cells, mitochondria, osteogenesis, salvianolic acid B

## Abstract

Long‐term or heavy use of glucocorticoids can cause severe necrosis of the femoral head, but the underlying mechanisms are still unclear. Recent studies have found that mitochondrial dynamics play an important role in femoral head necrosis. Here, we investigated the effect of dexamethasone on the mitochondrial function of mesenchymal stem cells. We observed that high concentrations of dexamethasone (10^−6^ mol·L^−1^) decreased cell activity, promoted apoptosis, elevated levels of reactive oxygen species and disrupted mitochondrial dynamics. Furthermore, dexamethasone (10^−6^ mol·L^−1^) inhibited osteogenesis of stem cells and promoted adipogenesis. These findings may facilitate greater understanding of the adverse effects of dexamethasone on the femoral head.

## Graphical abstract

Long‐term or heavy use of glucocorticoids can cause severe necrosis of the femoral head, but the underlying mechanisms are still unclear. Here, we found that high concentrations of dexamethasone can disrupt mitochondrial dynamics, which can cause increased mitochondrial division and decreased fusion, as well as enhanced osteogenesis and decreased adipogenesis of mesenchymal stem cells.

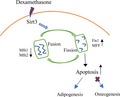

**Abbreviations**
ALP: alkaline phosphatase
C‐caspase‐3: cleaved caspase‐3
CCK‐8: Cell Counting Kit‐8
Cyt‐*c*: cytochrome *c*
Fis1: fission 1
HLA: human leukocyte antigen
MFF: mitochondrial fission factor
Mfn: mitofusin
MSC: mesenchymal stem cell
ONFH: osteonecrosis of the femoral head
PI: propidium iodide
ROS: reactive oxygen species
Sal B: salvianolic acid B
SD: standard deviation
SIRT: sirtuin
WB: western blotting

Osteonecrosis of the femoral head (ONFH) is a severe and high‐incidence disease in orthopedics that is closely related to excessive glucocorticoid use 1, 2. However, currently there is no effective clinical treatment for ONFH, and the reason is related to the unclear pathogenesis of ONFH. Bone marrow‐derived mesenchymal stem cells (MSCs) were multipotent progenitor cells that can differentiate into osteocytes, adipocytes, chondrocytes and so on 3. MSCs are important in the pathophysiology process of ONFH and have extraordinary multilineage differentiation capacity 4, 5. In recent years, the role of mitochondria in senescence‐related diseases has been widely concerning for domestic and foreign scholars. Mitochondria are not only the main energy supply center in cells, but also participate in the regulation of various cell signal transduction processes, such as Ca^2+^ homeostasis, cell apoptosis and production of reactive oxygen species (ROS) 6, 7. As a kind of highly dynamic network of tubular mitochondria organelles, through the continuous integration (fusion) and division (fission) to maintain their normal morphology and physiological function, the process is referred to as mitochondrial dynamics, which can repair the slightly damaged mitochondria; that is, mitochondrial fusion splits the proliferation and extends, which can realize the normal number of physiological conditions, integration and division in dynamic equilibrium 8. Mitochondrial kinetic imbalance and the ROS level increase induced by it play important roles in this process. The physiological level of ROS can act as an important second messenger in cells and participate in regulating cell growth, differentiation, metabolism and other activities. However, in the pathological state, the imbalance of mitochondrial kinetics and the induced increase in ROS level can lead to a variety of metabolic disorders. It has been confirmed that increased ROS levels also play an important role in BMSC osteogenic and lipogenic differentiation disorder 9, 10, 11. Sirtuins (SIRTs) are nicotinamide adenine dinucleotide^+^‐dependent deacetylases, belonging to the silencing information regulatory factor family, including different members of sirt1–7. Studies have shown that SIRTs are important in cell differentiation, death, aging, metabolism, inflammation, tumor and life span regulation, and other processes 12. *SIRT3* plays an important role in cell synthesis of nicotinamide adenine dinucleotide^+^, ATP and mitochondrial respiratory cycle, and is of great significance for the mitochondrial respiratory chain and antioxidant system 13, 14, 15, 16.

Salvianolic acid B (Sal B), a traditional Chinese compound, can protect neuro‐ and cardio‐correlative diseases 17, 18. Sal B is a famous antioxidant among the most effective natural products 19, 20.

Based on previous studies, we boldly speculated that glucocorticosteroid‐induced ONFH might lead to decreased osteogenesis and enhanced adipogenesis of MCSs by inducing mitochondrial fusion and division disorders of MSCs, and use of traditional Chinese medicine Sal B may effectively reduce apoptosis (Fig. 1).

**Figure 1:**
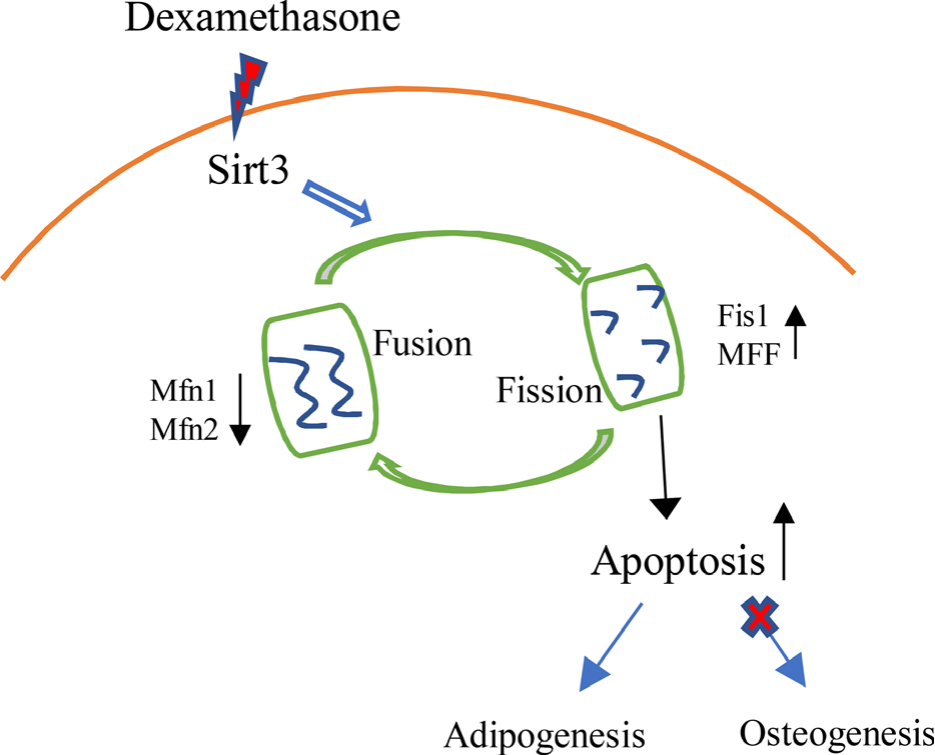
Schematic illustration of how dexamethasone induces decreased osteogenesis and increased adipogenesis by inducing mitochondrial dynamic disorders.

## Materials and methods

### MSCs

Human bone MSCs were harvested from the joint replacement of healthy volunteer donors. The donors signed the informed written consent. The study methodologies conformed to the standards set by the Declaration of Helsinki and approved by the ethics review committee of Union Hospital affiliated to Huazhong University of Science and Technology. Cells were cultured in Dulbecco’s modified Eagle’s medium–F12 containing 10% FBS and 1% penicillin–streptomycin. The 2–3 passage cells were used in the next experiments. Then we used flow cytometry (BD Biosciences, Franklin Lakes, NJ, USA) to detect the cell surface markers [CD73, CD90 and CD105 for positive expression and CD34 and human leukocyte antigen (HLA)‐DR for negative expression]. Moreover, the potential of MSCs for multilineage differentiation was determined in osteogenic, chondrogenic and adipogenic differentiation mediums, respectively (Cyagen, Suzhou, China).

### Cell Counting Kit‐8 assay

MSC viability was determined by Cell Counting Kit‐8 (CCK‐8; Dojindo, Tokyo, Japan) as described here. In brief, we first added 5000 cells per well in 96‐well culture plates. After 12 h, MSCs were treated with dexamethasone at various concentrations. At 1, 3 and 5 days later, the cells were cultured with 100 μL of Dulbecco’s modified Eagle’s medium–F12 containing 10% of CCK‐8 solution; then the plates were incubated in a cell incubator for 3 h. The absorbance was determined at 450 nm. Then the cell viability cultured with different concentrations (1, 5 and 10 μmol·mL^−1^) of Sal B was studied. For Sal B administration, Sal B was diluted to final concentrations of 1, 5 and 10 μm with serum‐free culture medium and then cultured for 3 days.

### Live/Dead staining

The effects of dexamethasone on MSCs were quantified by Calcein‐AM/propidium iodide (PI) (Dojindo). The samples were treated with different concentrations of dexamethasone for 3 days; then the cells were rinsed with PBS three times and incubated in PBS containing 2 μmol·L^−1^ Calcein‐AM and 4 μmol·L^−1^ PI in the dark for 15 min. The cells were then detected by a fluorescence microscope (Olympus, Tokyo, Japan) in the dark. Live cells showed green fluorescence, whereas the dead cells showed red fluorescence.

### Annexin V–PI staining

The MSCs from the experimental group and control group were harvested and washed with PBS; then the cells were labeled by Annexin V–PI double staining (KeyGen Biotech, Jiangsu, China). Ultimately, the samples were analyzed by flow cytometry. For Sal B administration, MSCs were cultured in a medium containing Sal B for 24 h before change to the different concentration of dexamethasone.

### Alizarin Red S, alkaline phosphatase staining and Oil Red O staining

After the MSCs were cultured in osteogenic induction medium in 24‐well culture plates with different concentrations of dexamethasone for 14 days, Alizarin Red S (Cyagen Biosciences, Inc., Santa Clara, CA, USA) was used according to the protocol. In brief, we used 4% paraformaldehyde to fix the cells. Then the cells were washed in PBS twice and treated with solution for 25 min, followed by washing with PBS and air‐drying. The samples were observed by an optical microscope.

For alkaline phosphatase (ALP) staining, after 14 days, the 4% paraformaldehyde was used to fix the MSCs for 20 min, then rinsed twice with PBS and incubated with ALP reagent in accordance to the manufacturer. The samples were observed by an optical microscope.

For Oil Red O staining, after 14 days, the 4% paraformaldehyde was used to fix the MSCs for 20 min, then rinsed twice with PBS and incubated with Oil Red O staining reagent. Then it was washed three times with PBS after 30 min. The samples were observed by an optical microscope.

### Electron microscopy

Cell samples for electron microscopic examination were first fixed by 2.5% glutaraldehyde at 8 h at 4 ℃; then the cells were rinsed three times with 0.1 m phosphate buffer (pH 7.4), each time for 15 min. One percent osmium tetroxide was used to fix samples for 2 h at room temperature; then they were washed again. After gradient dehydration using ethanol, the samples were embedded overnight. Finally, 80‐nm thin sections were stained and examined by transmission electronic microscopy (HT7700‐SS/FEI Tecnai G20 TWIN, FEI company, Hillsboro, OR, USA).

### Western blot analysis

Western blotting (WB) assays were used to test the proteins of indicator of mitochondrial fusion and division, including Sirt3, fission 1 (Fis1), mitochondrial fission factor (MFF), mitofusin 1 (Mfn1), mitofusin 2 (Mfn2), indicator for osteogenesis, adipogenesis [ALP, Runt‐related transcription factor 2, collagen type I, peroxisome proliferator‐activated receptor, adducin 1 (ADD1)] and mitochondrial‐apoptosis indicator [cleaved caspase‐3, cytochrome *c* (Cyt‐*c*)]. In brief, the MSCs were cultured with different concentrations of dexamethasone for 7 days. Proteins were extracted, and quantitation was performed by a BCA Protein Assay Kit (Beyotime, Shanghai, China). Equal amounts of protein from each group were separated by SDS/PAGE and then transferred to PVDF membranes (Millipore, Boston, MA, USA); before being incubated with primary antibodies, the membranes were blocked with 5% nonfat milk in TBST buffer and incubated at 4 °C overnight. Then we used TBST to wash the membranes three times and incubated the secondary antibody for 1 h. Finally, the protein expression was analyzed by AlphaEaseFC (IBM, Armonk, NY, USA). Glyceraldehyde‐3 phosphate dehydrogenase was used as normalization.

### Assessment of mitochondrial membrane potential

Mitochondrial membrane potential was detected by JC‐1 (C2006; Beyotime) assay kits according to our previous study 21. In brief, cells were cocultured with dexamethasone of different concentrations for 24 h; then the cells were centrifuged and collected. Jc‐1 working fluid was added and incubated in the cell culture box for 20 min. Finally, Jc‐1 buffer was used for washing twice and analyzed by flow cytometer (CytoFLEX; Beckman Coulter, Brea, CA, USA). Mitochondrial membrane potential loss was calculated according to the decrease ratio of red (JC‐1 aggregates) to green (JC‐1 monomers).

### Statistical analysis

Data are expressed as the mean values ± standard deviation (SD) of three independent experiments, and analysis was by spss 22.0. (IBM, Chicago, IL, USA). Differences between treatment groups and culture group were determined by Student’s *t*‐test or one‐way ANOVA. A *P*‐value < 0.05 was considered statistically significant.

## Results

### High concentration of dexamethasone inhibits cell activity

**Figure 2:**
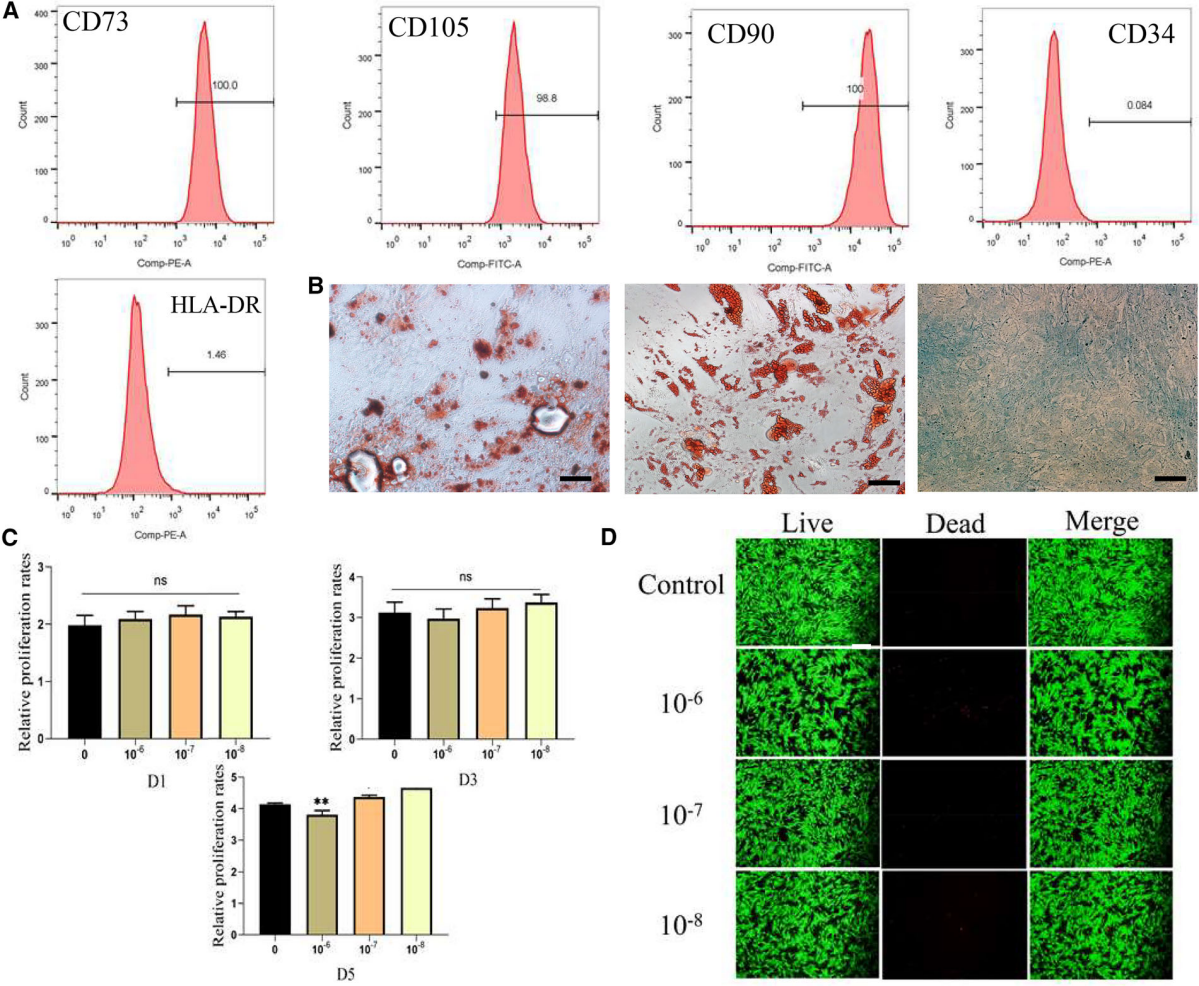
Isolation and identification of MSCs and CCK‐8 assay. (A) Cell surface markers (CD73, CD105, CD90, CD34 and HLA‐DR) of MSCs were detected by flow cytometric analysis. (B) The ability of MSCs to differentiate into the osteogenic, chondrogenic and adipogenic lineages was confirmed by Alizarin Red staining (left panel), Oil Red O staining (middle panel) and Alcian blue staining (right panel), respectively. (C) Cells were treated with 10^−6^, 10^−7^ and 10^−8^ mol·L^−1^ dexamethasone for 1, 3 and 5 days, followed by CCK‐8 assay to determine cell number/cell proliferation, with cells that received no treatment as the control. *n* = 3 per group. *P or **P represent significant difference (one‐way ANOVA). Values are represented as mean ± SD. *P < 0.01 (0.05), comparison between 10^−7^ mol·L^−1^ or 10^−8^ mol·L^−1^ with control; **P < 0.01, comparison between 10^−6^ mol·L^−1^. (D) Cells were treated with 10^−6^, 10^−7^ or 10^−8^ mol·L^−1^ dexamethasone for 72 h. Cell survival was monitored using Calcein‐AM/PI double staining. Scale bars: 50 μm.

Human MSCs were collected from the donors of joint replacement. Flow cytometry analysis indicated that MSC surface antigens were CD73^+^, CD105^+^ and CD90^+^, as well as CD34^−^ and HLA‐DR^−^ (Fig. 2A). Multilineage differentiation showed that MSCs can differentiate to osteogenesis, adipogenesis or chondrogenesis (Fig. 2B). We first explored the effect of different concentrations of dexamethasone on MSC activity. It was clear that the high concentration of dexamethasone (10^−6^ mol·L^−1^) inhibits the proliferation of MSCs, lower concentrations of dexamethasone promoted MSC proliferation (*P* < 0.05) after 5 days, and there is no statistical difference between the control group and experimental group after 1 and 3 days. As in most previous studies, high concentrations of dexamethasone have been shown to inhibit MSC proliferation and vice versa (Fig. 2C).

### Calcein‐AM/PI staining

The effects of different concentrations of dexamethasone on MSCs were quantified by Calcein‐AM/PI. Red cells increased with increasing dexamethasone concentrations. Obviously, more red signals appear at concentrations of 10^−6^ mol·L^−1^. These data implied that high concentrations of dexamethasone have cytotoxic effects on MSCs (Fig. 2D).

### High concentration of dexamethasone promotes apoptosis

**Figure 3:**
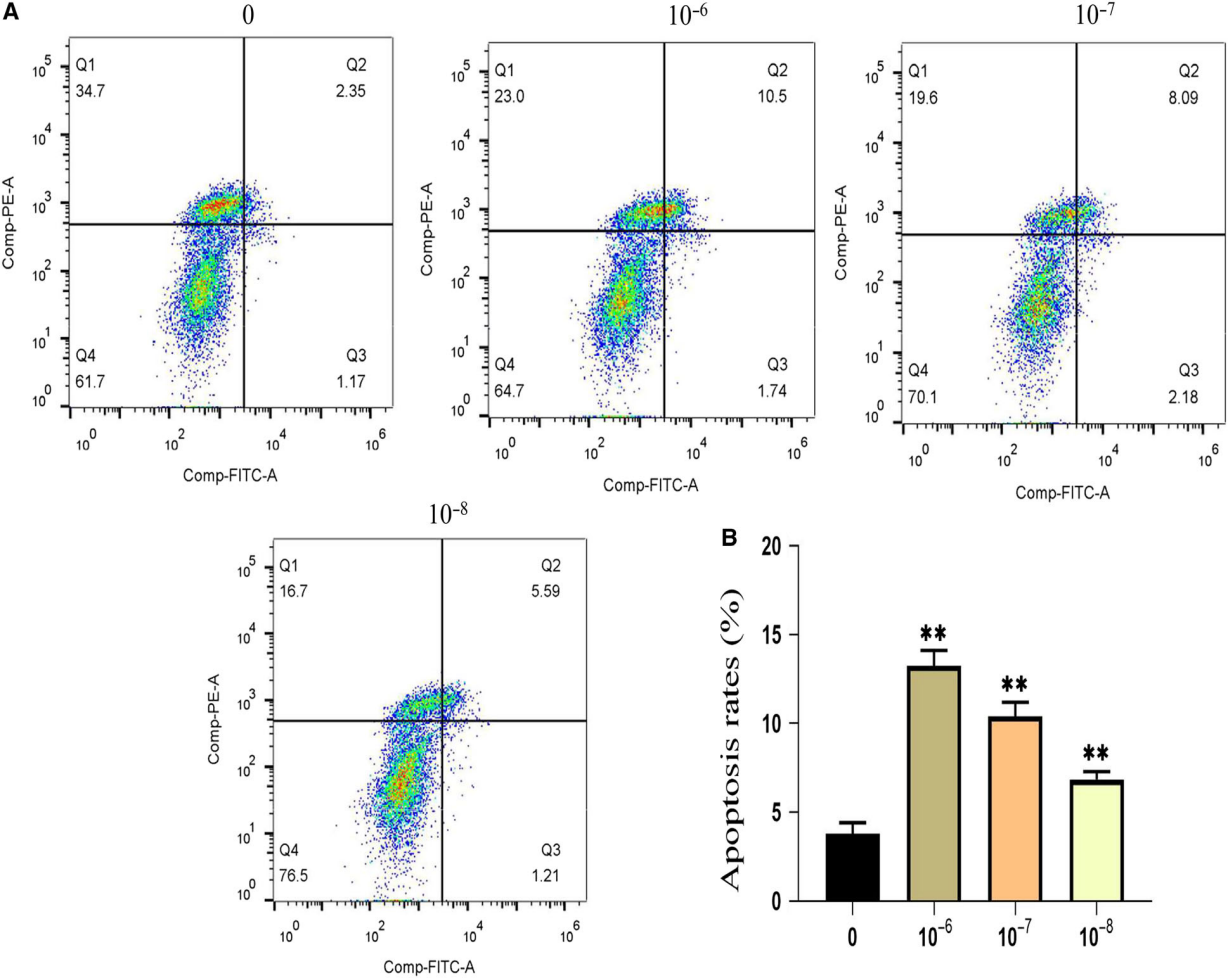
Role of dexamethasone on MSCs. (A, B) The representative scatterplots of Annexin V–PI staining and quantitative analysis of apoptosis ratio. *n* = 3 per group. **P represent significant difference (one‐way ANOVA). Values are represented as mean ± SD. **P < 0.01 versus with the control group.

To investigate whether a high concentration of dexamethasone induces MSC apoptosis, we applied Annexin V–PI staining to exploited apoptosis. Concentrations of 10^−6^ mol·L^−1^ dexamethasone significantly induced MSC apoptosis compared with control cells (Fig. 3A). Further quantitative results showed that dexamethasone treatment had a dose‐dependent increase in cell apoptosis (Fig. 3B).

### Dexamethasone increases mitochondrial division and reduces fusion

**Figure 4:**
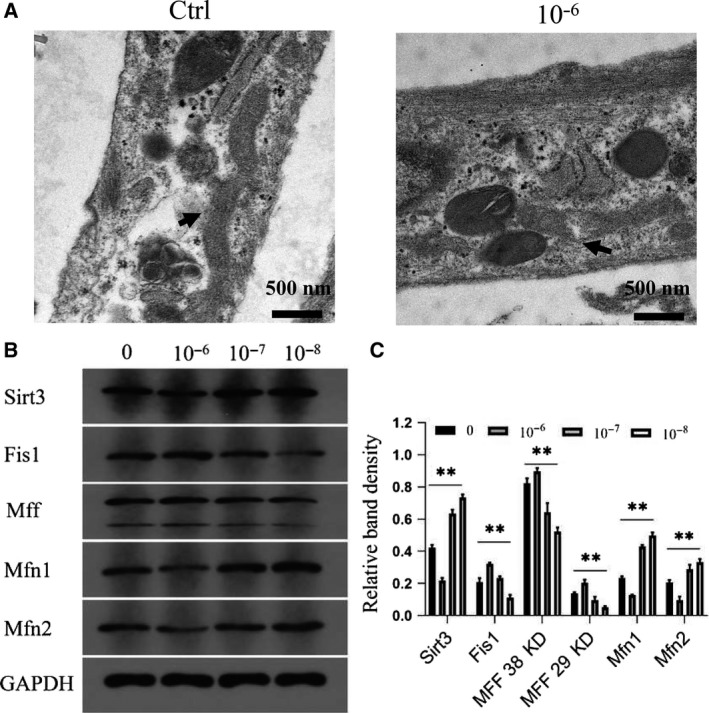
Dexamethasone increases mitochondrial division and reduces fusion. (A) Electron micrographs of the control group and 10^−6^ mol·L^−1^ dexamethasone group. (B, C) Representative WB assay and quantitation of the levels of Sirt3, Fis1, MFF, Mfn1 and Mfn2 in mitochondrial extracts. *n* = 3 per group. **P represent significant difference (one‐way ANOVA). Values are represented as mean ± SD. **P < 0.01 versus with the control group. Scale bars: 500 nm.

Changes in mitochondrial homeostasis will lead to changes in cell function. First, as shown in Fig. 4A, complete mitochondrial structure and mitochondrial cristae can be seen in the control group; in contrast, in the high concentration of dexamethasone group, the fission of mitochondria and the disappearance of mitochondrial cristae were clearly evident. Then, WB was used to verify changes in mitochondrial function after the intervention of different concentrations of dexamethasone. Sirt3, mitochondrial fusion indicators Mfn1 and Mfn2, and mitochondrial division indicators Fis1 and MFF were detected. As shown in Fig. 4B,C, with the increase of dexamethasone concentration, all fusion indictors decreased, fission indicators increased and Sirt3 declined gradually.

### The mitochondrial pathway was involved in dexamethasone‐induced MSC apoptosis

**Figure 5:**
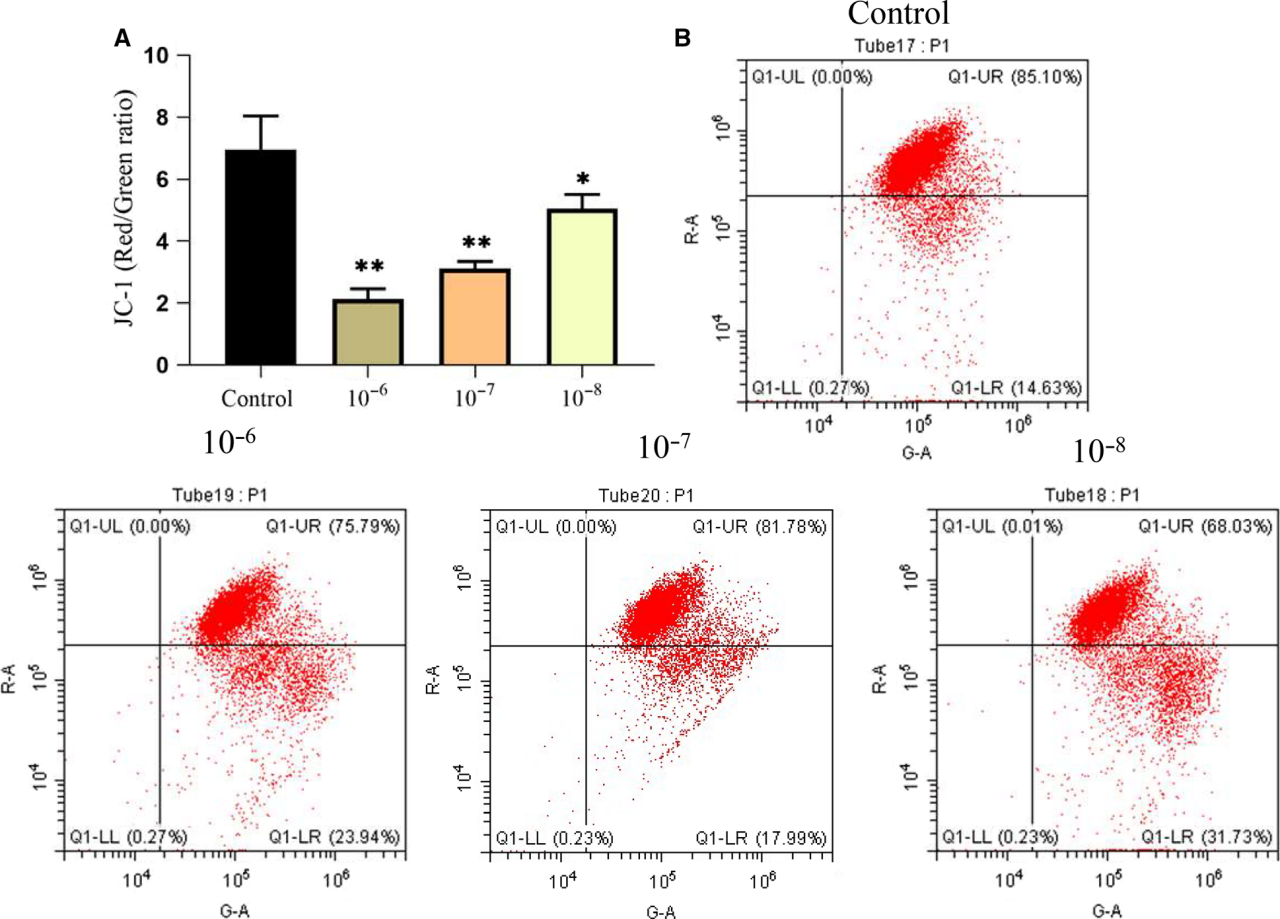
Dexamethasone treatment induced mitochondrial dysfunction. (A, B) The quantitative analysis of the shift of red fluorescence (*x*‐axis) to green fluorescence (*y*‐axis) for mitochondrial membrane potential and representative scatterplots of flow cytometry by JC‐1 staining. *n* = 3 per group. *P or **P represent significant difference (one‐way ANOVA). Values are represented as mean ± SD. **P < 0.01, *P < 0.05 versus control.

**Figure 6:**
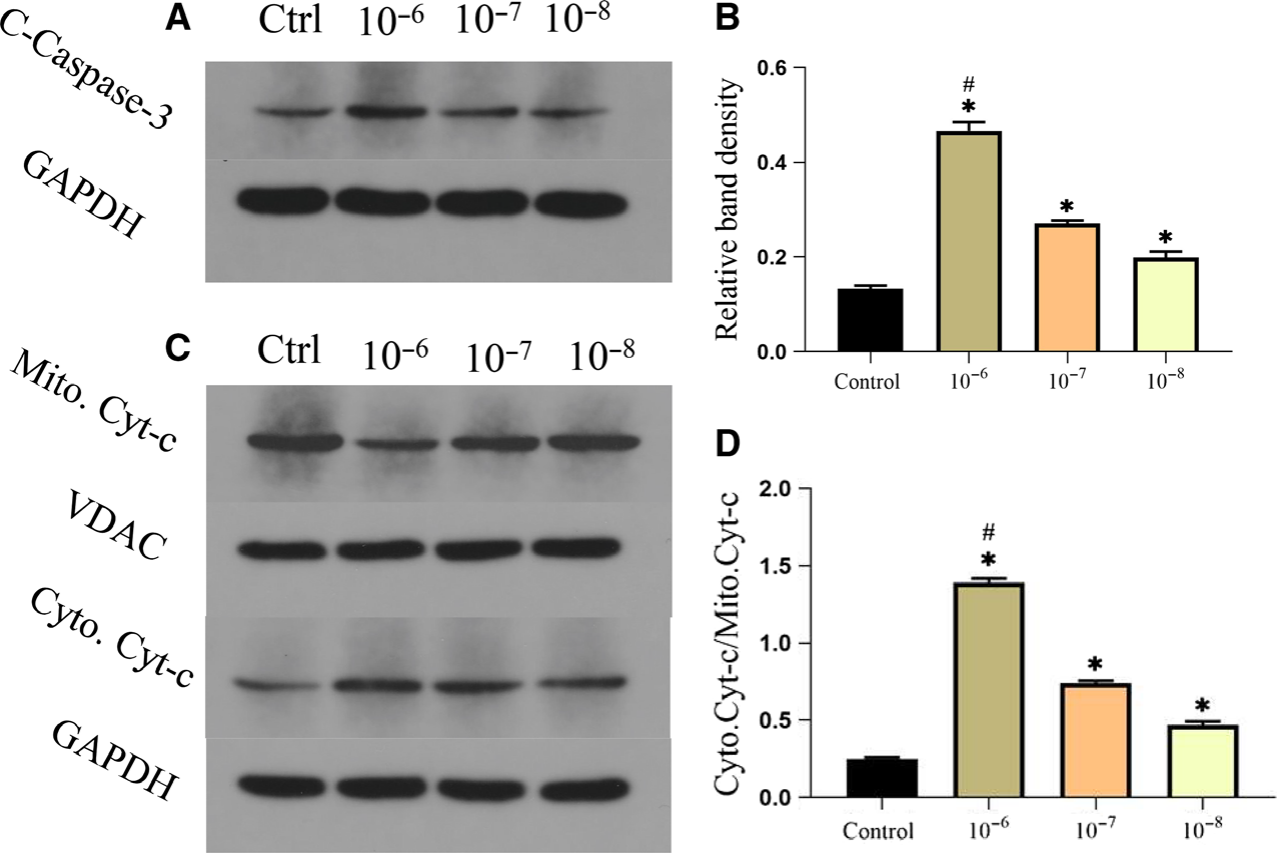
Dexamethasone promotes MSC apoptosis. (A, B) Representative WB assay and quantitation of the level of C‐caspase‐3. (C, D) Representative WB assay and quantitation of the level of Cyt‐*c*. *n* = 3 per group. *P represent significant difference (one‐way ANOVA). Values are represented as mean ± SD. *P < 0.05 versus with the control group; ^#^ *P* < 0.05 versus between 10^−6^ and 10^−7^ mol·L^−1^ dexamethasone groups.

The cell apoptotic events can be regulated through the mitochondrial pathway, which mainly contains mitochondrial membrane permeabilization and proapoptotic proteins. As shown in Fig. 5A,B, dexamethasone treatment enhanced the mitochondrial membrane potential loss in MSCs. Then, we examined the proapoptotic proteins including cleaved caspase‐3 (C‐caspase‐3) and Cyt‐*c*. As shown in Fig. 6A,B, compared with the control group, C‐caspase‐3 in the concentration of 10^−6^ mol·L^−1^ dexamethasone group increased significantly, and there was a significant statistical difference (*P* < 0.05); the comparison between the 10^−6^ and 10^−7^ mol·L^−1^ dexamethasone groups also showed a significant statistical difference (*P* < 0.05). Finally, we examined the Cyt‐*c* location in the mitochondria and the cytoplasm. WB analysis has shown that the ratio of cytoplasmic to mitochondrial Cyt‐*c* was significantly increased by dexamethasone treatment (Fig. 6C,D). These data revealed that dexamethasone affected the mitochondrial apoptotic pathway.

### Dexamethasone increases adipogenesis and inhibits osteogenesis

**Figure 7:**
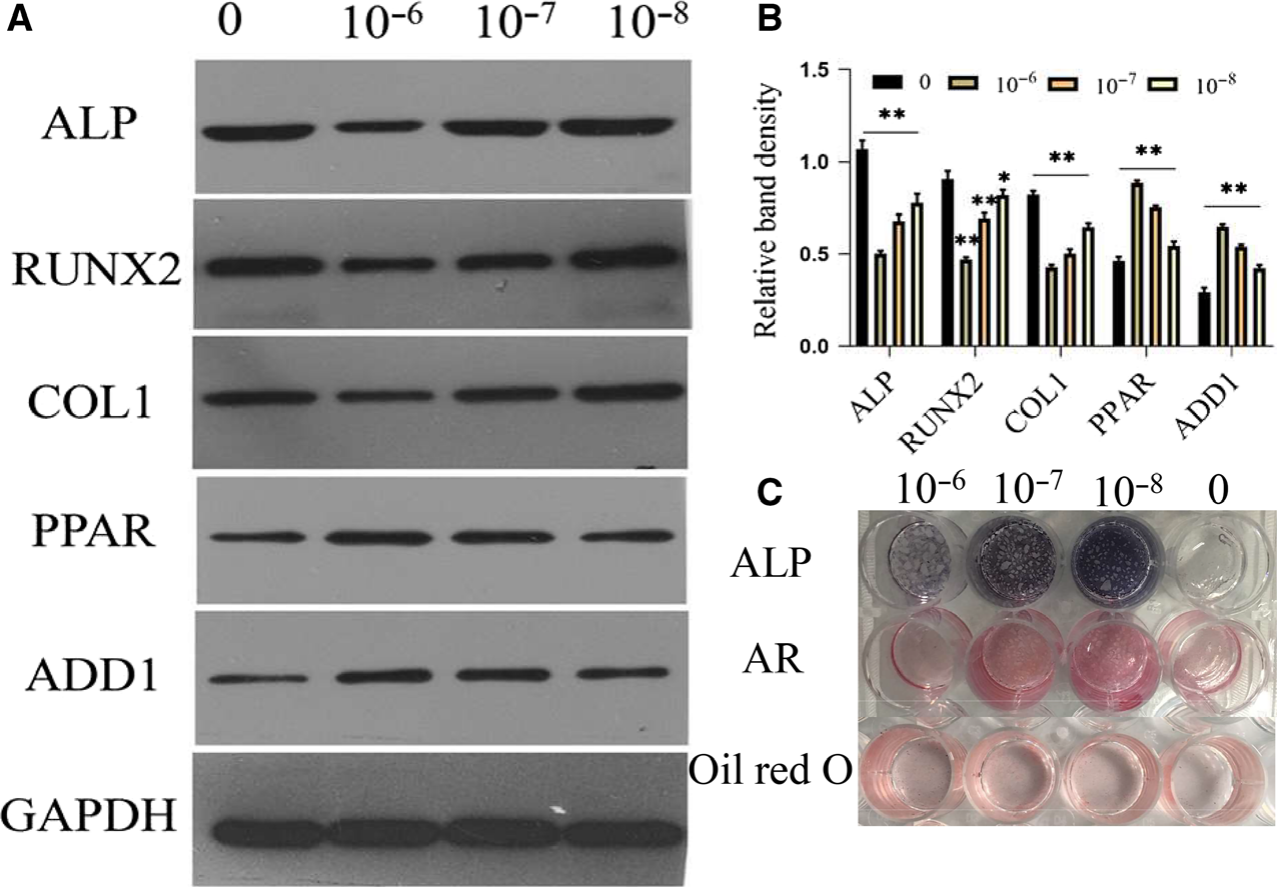
Dexamethasone increases adipogenesis and inhibits osteogenesis. (A, B) Representative WB assay and quantitation of the levels of ALP, Runt‐related transcription factor 2 (RUNX2), Col1, peroxisome proliferator‐activated receptor (PPAR) and adducin 1 (ADD1) in cytoplasmic extracts. *n* = 3 per group. *P or **P represent significant difference (one‐way ANOVA). Values are represented as mean ± SD. *P < 0.05, **P < 0.01 versus with the control group. (C) Representative images of Alizarin Red, ALP and Oil Red O staining of 10^−6^, 10^−7^ and 10^−8^ mol·L^−1^ dexamethasone on MSCs for 14 days.

We used WB to verify the effect of dexamethasone on osteogenesis and adipogenesis. As shown in Fig. 7A,B, compared with the control group, with the increase of dexamethasone concentration, the osteogenic indicators decreased and the adipogenic indicators increased. To further investigate the effect of different concentrations of dexamethasone on MSC osteogenesis, 14 days after osteogenic induction, we performed Alizarin Red, ALP and Oil Red O staining. As shown in Fig. 7C, with the increase of dexamethasone concentration, the osteogenic potential decreases and adipogenesis increases.

### Sal B effect on MSC apoptosis

**Figure 8:**
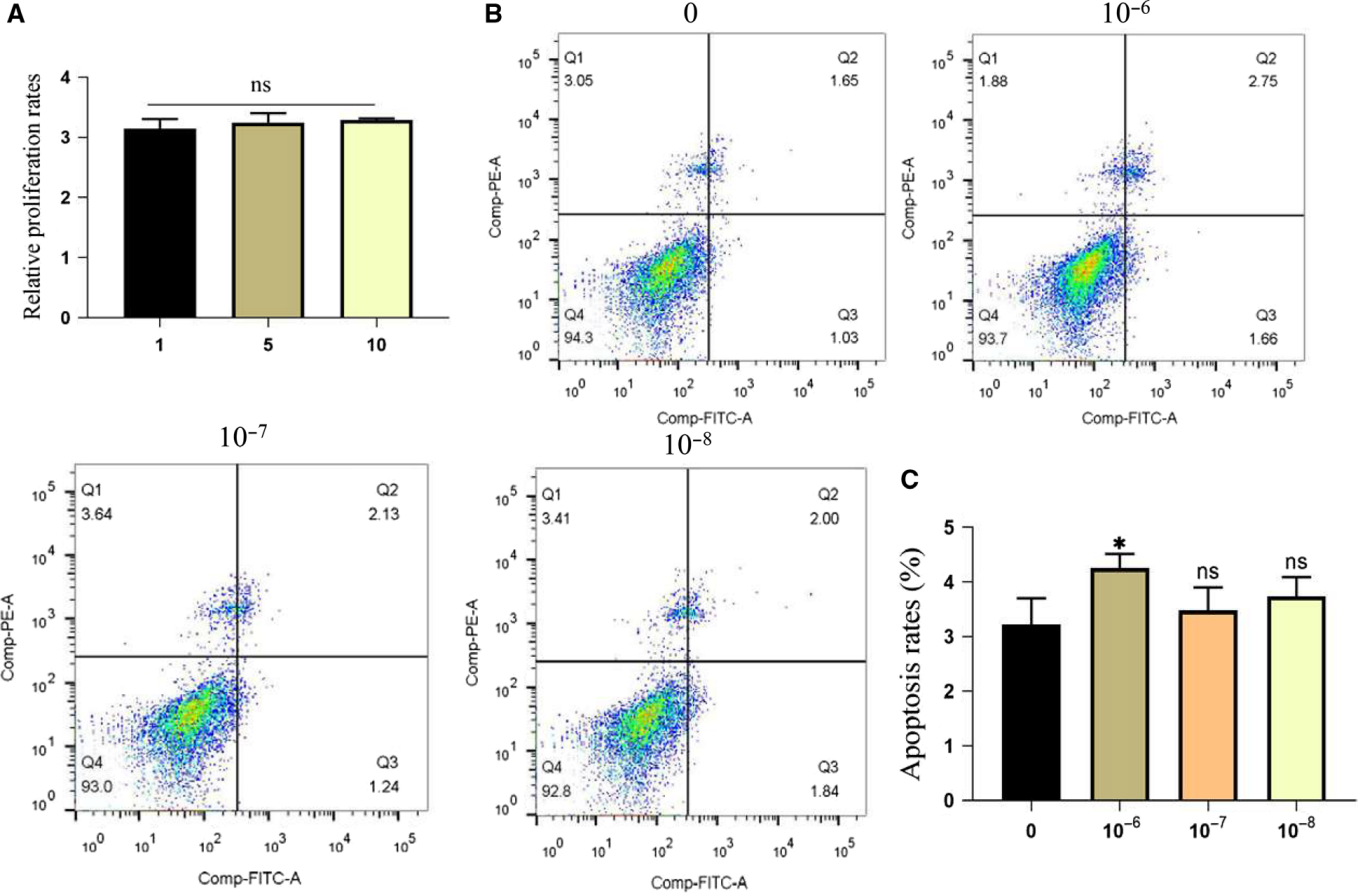
The role of Sal B on MSCs. (A) Cells were treated with 1, 5 and 10 μm Sal B for 3 days followed by CCK‐8 assay to determine cell number/cell proliferation, with cells that received no treatment as the control. (B, C) Cells were treated with 10 μm Sal B for 24 h, then washed with PBS, incubated with the different concentrations of dexamethasone and analyzed by Annexin V–PI staining. *n* = 3 per group. *P represent significant difference (one‐way ANOVA). Values are represented as mean ± SD. *P < 0.05 versus with control group. ns, not significant with control group.

We first investigated the effects of different concentrations of Sal B on MSC activity. As shown in Fig. 8A, cell viability was not significantly different (*P* > 0.05). Then we investigated whether Sal B can inhibit dexamethasone‐induced apoptosis. Although Sal B can inhibit apoptosis in the 10^−7^ and 10^−8^ mol·L^−1^ dexamethasone groups, there were still statistically differences between the control and 10^−6^ mol·L^−1^ dexamethasone groups (Fig. 8B,C).

## Discussion

Oxidative stress is important in the pathophysiology of many diseases, such as atherosclerosis, hypertension, diabetes and heart failure 22, 23, 24. A current study found that glucocorticoid treatment can decrease osteogenesis and increase adipogenesis, because both osteogenesis and adipogenesis begin in the bone marrow precursor cells 25. Mitochondria are not only the main energy supply center in cells, but also participate in the regulation of various cell signal transduction processes, such as Ca^2+^ homeostasis, cell apoptosis and production of ROS.

However, it has not been reported whether the mitochondrial dynamics disorder will lead to the changes of osteogenesis and adipogenesis in MSCs. First, we investigated the effects of dexamethasone at different concentrations on cell activity by CCK‐8 assay. We found that a high concentration of dexamethasone (10^−6^ mol·L^−1^) significantly reduced cell activity after 5 days. Then we used Live/Dead assay to further detect the cell viability and as with the CCK‐8 assay, more dead cells were shown in 10^−6^ mol·L^−1^ groups after 3 days. To quantitatively detect apoptosis rate, we used Annexin V–PI staining. More than 12.5 ± 1.2% of cells were apoptotic. We then examined mitochondrial fusion and division by electron micrographs and WB. As expected, mitochondrial division increased and fusion decreased when the concentration of dexamethasone increased. Next, we evaluated the mitochondrial membrane potential and the related indicators of apoptosis; we found that high concentrations of dexamethasone can cause loss of mitochondrial membrane potential and promote apoptosis. We then looked at whether mitochondrial dynamics disorder could lead to increased osteogenesis and decreased adipogenesis. Alizarin Red, ALP and Oil Red O staining showed that osteogenesis reduced significantly and adipogenesis increased after 14 days of culture with different concentration of dexamethasone. WB assay results further confirmed that a high concentration of dexamethasone resulted in decreased osteogenesis and increased adipogenesis. Then we examined whether Sal B can inhibit apoptosis. Flow cytometry showed that Sal B can inhibit apoptosis in the 10^−7^ and 10^−8^ mol·L^−1^ dexamethasone groups. Lu *et al*. 26 reported that Sal B can protect rat MSCs against oxidative stress‐induced apoptosis, indicating that Sal B may reduce ROS production by protecting dexamethasone‐induced mitochondrial disorders, thereby promoting the transformation of MSCs into osteoblasts. As shown in our group, although Sal B can inhibit apoptosis in the 10^−7^ and 10^−8^ mol·L^−1^ dexamethasone groups, there were still statistical differences between control and 10^−6^ mol·L^−1^ dexamethasone groups. Sal B had no significant effect on apoptosis induced by dexamethasone in our study.

However, our study still has some limitations. Although our study found that a high concentration of dexamethasone can induce apoptosis by dynamics disorders of mitochondria, further investigation is needed to fully reveal the underlying mechanisms in dexamethasone modulation of mitochondria disorder. In addition, the protective effect of Sal B on mitochondria needs to be further explored. In summary, high concentrations of dexamethasone can cause mitochondrial dynamics disorders that can lead to increased mitochondrial division and decreased fusion, and MSCs osteogenesis increases and adipogenesis decreases.

## Conflict of interest

The authors declare no conflict of interest.

## Author contributions

LM, XF and CY designed experiments. LM and KW carried out experiments. XF, KW and YS analyzed experimental results. LM and RL wrote the manuscript. LM, XF and CY assisted with the proof.

## References

[1] Luo P , Gao F , Han J , Sun W and Li Z (2018) The role of autophagy in steroid necrosis of the femoral head: a comprehensive research review. Int Orthop 42, 1747–1753. 29797168 10.1007/s00264-018-3994-8

[2] Liu LH , Zhang QY , Sun W , Li ZR and Gao FQ (2017) Corticosteroid‐induced osteonecrosis of the femoral head: detection, diagnosis, and treatment in earlier stages. Chin Med J (Engl) 130, 2601–2607. 29067959 10.4103/0366-6999.217094 PMC5678261

[3] Cui P , Zhao X , Liu J , Chen X , Gao Y , Tao K , Wang C and Zhang X (2019) miR‐146a interacting with lncRNA EPB41L4A‐AS1 and lncRNA SNHG7 inhibits proliferation of bone marrow‐derived mesenchymal stem cells. J Cell Physiol. 10.1002/jcp.29217 PMC13482691 31612476

[4] Keating A (2012) Mesenchymal stromal cells: new directions. Cell Stem Cell 10, 709–716. 22704511 10.1016/j.stem.2012.05.015

[5] Wang X , Wang Y , Gou W , Lu Q , Peng J and Lu S (2013) Role of mesenchymal stem cells in bone regeneration and fracture repair: a review. Int Orthop 37, 2491–2498. 23948983 10.1007/s00264-013-2059-2 PMC3843208

[6] Gavish M and Veenman L (2018) Regulation of mitochondrial, cellular, and organismal functions by TSPO. Adv Pharmacol 82, 103–136. 29413517 10.1016/bs.apha.2017.09.004

[7] Vafai SB and Mootha VK (2012) Mitochondrial disorders as windows into an ancient organelle. Nature 491, 374–383. 23151580 10.1038/nature11707

[8] Wai T and Langer T (2016) Mitochondrial dynamics and metabolic regulation. Trends Endocrinol Metab 27, 105–117. 26754340 10.1016/j.tem.2015.12.001

[9] Higuchi M , Dusting GJ , Peshavariya H , Jiang F , Hsiao ST , Chan EC and Liu GS (2013) Differentiation of human adipose‐derived stem cells into fat involves reactive oxygen species and Forkhead box O1 mediated upregulation of antioxidant enzymes. Stem Cells Dev 22, 878–888. 23025577 10.1089/scd.2012.0306 PMC3585477

[10] Almeida M , Han L , Martin‐Millan M , O'Brien CA and Manolagas SC (2007) Oxidative stress antagonizes Wnt signaling in osteoblast precursors by diverting beta‐catenin from T cell factor‐ to forkhead box O‐mediated transcription. J Biol Chem 282, 27298–27305. 17623658 10.1074/jbc.M702811200

[11] Hofmann AD , Beyer M , Krause‐Buchholz U , Wobus M , Bornhauser M and Rodel G (2012) OXPHOS supercomplexes as a hallmark of the mitochondrial phenotype of adipogenic differentiated human MSCs. PLoS ONE ONE 7, e35160. 10.1371/journal.pone.0035160 PMC3327658 22523573

[12] Lombard DB and Zwaans BM (2014) SIRT3: as simple as it seems? Gerontology 60, 56–64. 24192814 10.1159/000354382 PMC3875292

[13] Lombard DB , Alt FW , Cheng H‐L , Bunkenborg J , Streeper RS , Mostoslavsky R , Kim J , Yancopoulos G , Valenzuela D , Murphy A * et al* (2007) Mammalian Sir2 homolog SIRT3 regulates global mitochondrial lysine acetylation. Mol Cell Biol 27, 8807–8814. 17923681 10.1128/MCB.01636-07 PMC2169418

[14] Samant SA , Zhang HJ , Hong Z , Pillai VB , Sundaresan NR , Wolfgeher D , Archer SL , Chan DC and Gupta MP (2014) SIRT3 deacetylates and activates OPA1 to regulate mitochondrial dynamics during stress. Mol Cell Biol 34, 807–819. 24344202 10.1128/MCB.01483-13 PMC4023816

[15] Morigi M , Perico L , Rota C , Longaretti L , Conti S , Rottoli D , Novelli R , Remuzzi G and Benigni A (2015) Sirtuin 3‐dependent mitochondrial dynamic improvements protect against acute kidney injury. J Clin Invest 125, 715–726. 25607838 10.1172/JCI77632 PMC4319434

[16] Song W , Song Y , Kincaid B , Bossy B and Bossy‐Wetzel E (2013) Mutant SOD1G93A triggers mitochondrial fragmentation in spinal cord motor neurons: neuroprotection by SIRT3 and PGC‐1alpha. Neurobiol Dis 51, 72–81. 22819776 10.1016/j.nbd.2012.07.004 PMC3992938

[17] Tang MK , Ren DC , Zhang JT and Du GH (2002) Effect of salvianolic acids from Radix Salviae miltiorrhizae on regional cerebral blood flow and platelet aggregation in rats. Phytomedicine 9, 405–409. 12222659 10.1078/09447110260571634

[18] Zhang HS and Wang SQ (2006) Salvianolic acid B from *Salvia miltiorrhiza* inhibits tumor necrosis factor‐alpha (TNF‐alpha)‐induced MMP‐2 upregulation in human aortic smooth muscle cells via suppression of NAD(P)H oxidase‐derived reactive oxygen species. J Mol Cell Cardiol 41, 138–148. 16713603 10.1016/j.yjmcc.2006.03.007

[19] Adams JD , Wang R , Yang J and Lien EJ (2006) Preclinical and clinical examinations of *Salvia miltiorrhiza* and its tanshinones in ischemic conditions. Chin Med 1, 3. 17302964 10.1186/1749-8546-1-3 PMC1761145

[20] Li YG , Song L , Liu M , Hu ZB and Wang ZT (2009) Advancement in analysis of *Salviae miltiorrhizae* Radix et Rhizoma (Danshen). J Chromatogr A 1216, 1941–1953. 19159889 10.1016/j.chroma.2008.12.032

[21] Song Y , Li S , Geng W , Luo R , Liu W , Tu J , Wang K , Kang L , Yin H , Wu X * et al* (2018) Sirtuin 3‐dependent mitochondrial redox homeostasis protects against AGEs‐induced intervertebral disc degeneration. Redox Biol 19, 339–353. 30216853 10.1016/j.redox.2018.09.006 PMC6139007

[22] Leopold JA and Loscalzo J (2005) Oxidative enzymopathies and vascular disease. Arterioscler Thromb Vasc Biol 25, 1332–1340. 15790928 10.1161/01.ATV.0000163846.51473.09

[23] Davì G and Falco A (2005) Oxidant stress, inflammation and atherogenesis. Lupus 14, 760–764. 16218483 10.1191/0961203305lu2216oa

[24] Cai H and Harrison DG (2000) Endothelial dysfunction in cardiovascular diseases: the role of oxidant stress. Circ Res 87, 840–844. 11073878 10.1161/01.res.87.10.840

[25] Blair HC , Sun L and Kohanski RA (2007) Balanced regulation of proliferation, growth, differentiation, and degradation in skeletal cells. Ann N Y Acad Sci 1116, 165–173. 17646258 10.1196/annals.1402.029

[26] Lu B , Ye Z , Deng Y , Wu H and Feng J (2010) MEK/ERK pathway mediates cytoprotection of salvianolic acid B against oxidative stress‐induced apoptosis in rat bone marrow stem cells. Cell Biol Int 34, 1063–1068. 20629637 10.1042/CBI20090126

